# A striking case of cutaneous adult T-cell leukemia/lymphoma mimicking subacute cutaneous lupus erythematosus

**DOI:** 10.1016/j.jdcr.2025.01.045

**Published:** 2025-06-24

**Authors:** Daniel S. Alicea, Suraj Muddasani, Michael Occidental, Bijal Amin, Beth N. McLellan, Benedict Wu

**Affiliations:** Division of Dermatology, Department of Medicine, Albert Einstein College of Medicine, Montefiore Medical Center, Bronx, New York

**Keywords:** cutaneous T-cell leukemia, cutaneous T-cell lymphoma, human T-cell lymphotropic virus, oncodermatology, subacute cutaneous lupus erythematosus

*To the Editor:* We appreciated the article from Cohen et al,^1^ which discussed how adult T-cell lymphoma (ATLL) may mimic other types of lymphomas, such as cutaneous T-cell lymphoma (CTCL). Skin involvement in ATLL cases may be present before (defined as skin-first), at, or after diagnosis (defined as skin-second). More than 80% of ATLL patients with skin-first involvement have a preceding diagnosis of CTCL.^2^ Cutaneous manifestations of ATLL are heterogeneous and rarely present in sun-exposed sites. This makes the diagnosis and workup challenging as ATLL may clinically appear similar to other inflammatory dermatoses, such as subacute cutaneous lupus erythematosus (SCLE). Herein, we describe a case of skin-first ATLL clinically mimicking SCLE due to the annular scaly plaques in photo-exposed sites, thereby expanding the clinical phenotype of ATLL.

A 67-year-old woman of Caribbean descent presented with an asymptomatic but worsening skin eruption for the past 4 months. The patient was treated with topical steroids without improvement. She denied systemic symptoms and reported no new or changing lesions. Examination revealed scaly red to violaceous annular and polycyclic papules and plaques on the face, forearms, chest, upper back, and legs (Fig 1). The cervical, axillary, and inguinal node examination was negative for lymphadenopathy. The differential diagnoses included tinea corporis, SCLE, gyrate erythemas, and cutaneous lymphomas. A potassium hydroxide preparation was negative for tinea.

**Fig 1:**
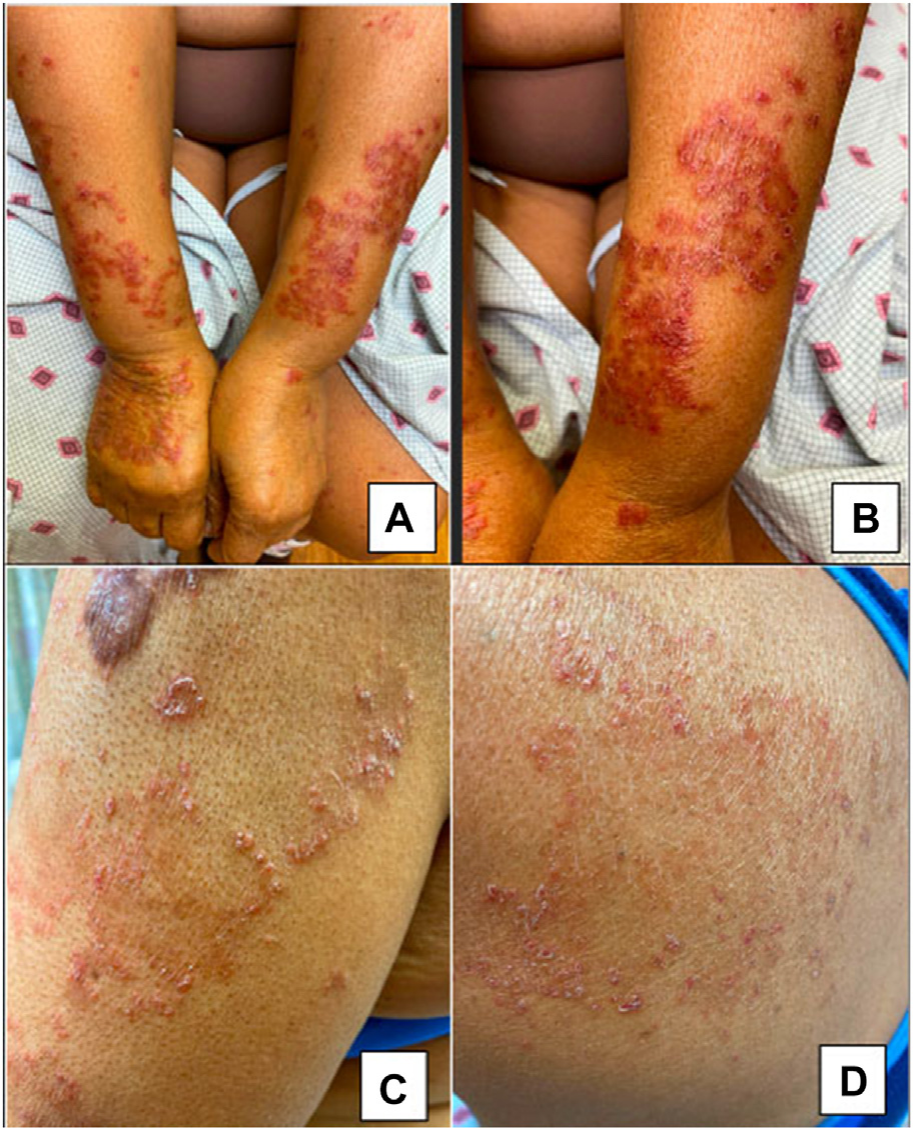
Scaly red to violaceous annular papules and plaques on (**A**, **B**) the bilateral extensor forearms, (**C**) lateral aspect of the upper portion of the arm, and (**D**) lateral upper back.

An outside biopsy reported an atypical CD4^+^ lymphoid infiltrate, concerning for CTCL; however, given the progression of the eruption and unusual clinical morphologies for CTCL, a repeat punch biopsy was performed from the right forearm, which showed an atypical epidermotropic lymphocytic infiltrate that was positive for CD2, CD3, CD4, or CD25 with loss of CD7, which raised additional concern for ATLL (Fig 2). The patient’s serum tested positive for the human T-lymphotropic virus type I (HTLV-1) antibody with negative antinuclear and antiextractable nuclear antigen antibodies, including anti-Ro(SS-A) antibody. Subsequent evaluation by oncology with a positron emission tomography-computed tomography scan confirmed superficial and deep nodal involvement above and below the diaphragm and spleen with an abnormal T-cell population on peripheral and bone marrow flow cytometry, supporting the diagnosis of systemic ATLL with cutaneous involvement.

**Fig 2:**
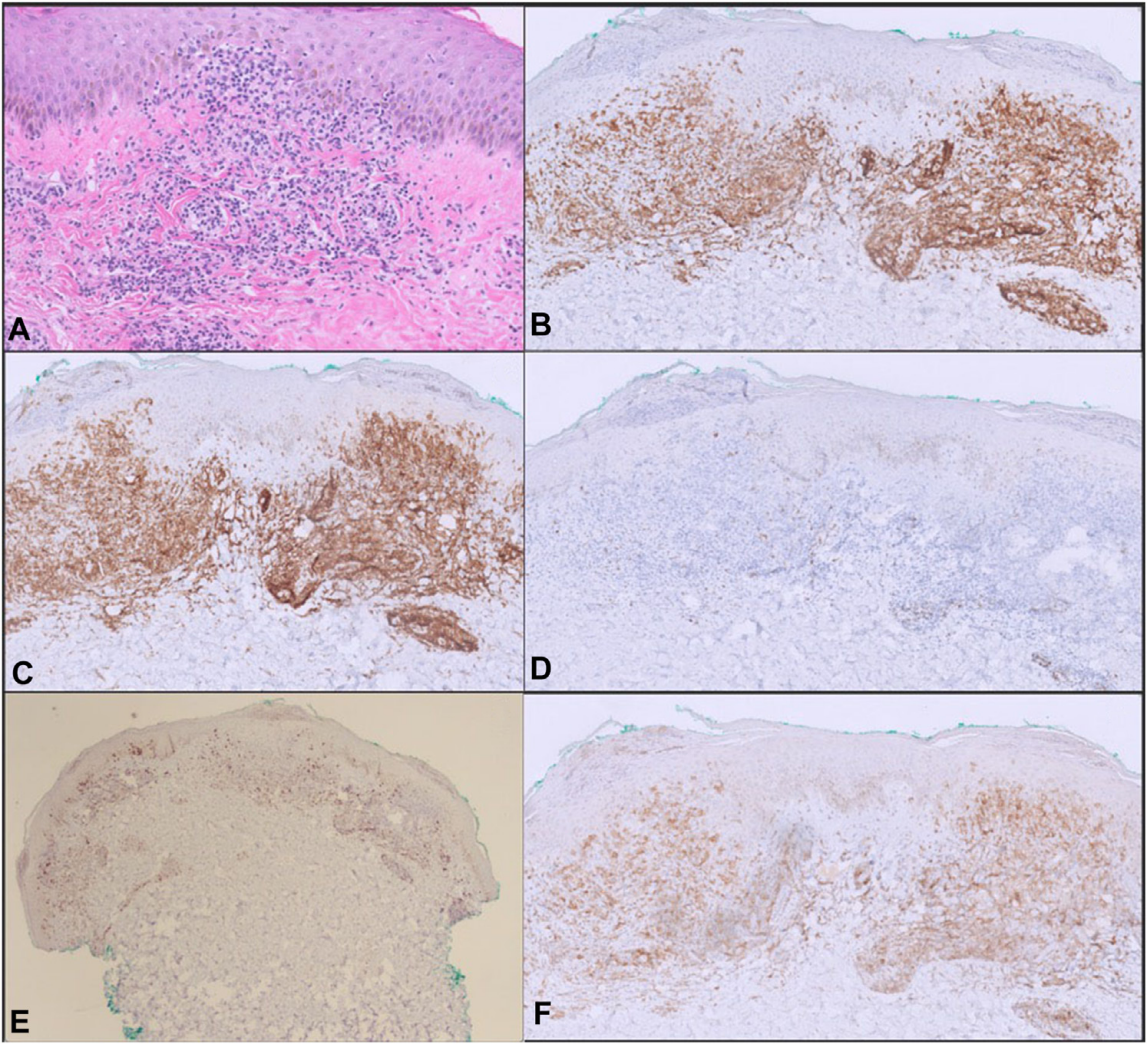
(**A**) Inflammatory infiltrates of enlarged and atypical lymphocytes with extension into the epidermis (**A**, Hematoxylin-eosin stain; original magnification: A, ×20). (**B**) CD3^+^, (**C**) CD4^+^, (**D**) CD7^−^, (**E**) CD8^+^, and (**F**) CD25^+^ expression within the atypical lymphocytic infiltrate, concerning for adult T-cell leukemia or lymphoma. (**A,** Hematoxylin-eosin stain; original magnification: **A,** ×20).

Our patient represents an unusual presentation of a SCLE-like ATLL. Approximately 50% of patients with ATLL have heterogeneous skin manifestations such as diffuse erythema, erythroderma, papular rashes, patches, plaques, nodules, purpura, or combinations of these findings, usually in nonphoto-distributed areas.^3^^,^^4^ This case highlights the importance of including cutaneous lymphomas in the differential diagnosis of a new rash, even if it is photo-distributed. Additionally, this case demonstrates the importance of checking HTLV-1 antibodies in cases mimicking CTCL, especially in patients from HTLV-endemic areas, as histopathologic findings can be identical. ATLL and CTCL share CD3^+^, CD4^−^, or CD7^−^ immunophenotypes and CD25 positivity is not definitive. HTLV-1 antibody testing is essential to differentiate ATLL from CTCL. To our knowledge, a SCLE-like presentation of ATLL has not been previously reported, and we urge clinicians to maintain a high index of suspicion of ATLL or other CTCL in patients presenting with annular scaly plaques in sun-exposed sites.

## Conflicts of interest

None disclosed.
